# Unintentional benzodiazepine use and frequency of drug checking service utilization: a cross-sectional study

**DOI:** 10.1186/s12954-025-01381-y

**Published:** 2026-01-20

**Authors:** Lauren Airth, Trevor Goodyear, Brandon D. L. Marshall, Cameron Grant, Mark Lysyshyn, Susan G. Sherman, Evan Wood, Lianping Ti

**Affiliations:** 1 British Columbia Centre On Substance Use, 400-1045 Howe Street, Vancouver, BC V6Z 2A9 Canada; 2 School of Nursing, University of British Columbia, 1147 Research Road, Kelowna, BC V1V 1V7 Canada; 3 Campus Wellness and Education, University of British Columbia, 3272 University Way, Kelowna, BC V1V 1V7 Canada; 4 School of Nursing, University of British Columbia, T201-2211 Wesbrook Mall, Vancouver, BC V6T 2B5 Canada; 5 Department of Epidemiology, Brown University School of Public Health, 121 S Main St, Providence, Rhode Island 02903 USA; 6 Vancouver Coastal Health, 600-601 West Broadway, Vancouver, BC V5Z 4C2 Canada; 7 School of Population and Public Health, University of British Columbia, 2206 E Mall, Vancouver, BC V6T 1Z3 Canada; 8 Department of Health, Behavior and Society, Johns Hopkins Bloomberg School of Public Health, 624 N Broadway Street, Baltimore, MD 21205 USA; 9 Department of Medicine, University of British Columbia, 2775 Laurel Street, Vancouver, BC V6H 0A5 Canada

**Keywords:** Harm reduction, Drug checking, Unregulated drugs, Benzodiazepines, Gender, Public health

## Abstract

**Background:**

There are increasing accounts of benzodiazepine adulteration and contamination in the unregulated drug supply in Canada and internationally. In some Canadian settings, drug checking services (DCS) are available for people to identify the constituents of their drugs. This study examines the relationship between suspected, unintentional benzodiazepine consumption and DCS utilization frequency, and whether gender modifies this relationship. We hypothesized that suspected unintentional benzodiazepine consumption would predict frequent DCS utilization, particularly for women, who may be at heightened risk for pertinent harms related to benzodiazepine use.

**Methods:**

Data were drawn from a cross-sectional study (2021–2023) evaluating DCS that used spectroscopy and immunoassay strips in British Columbia, Canada. Multivariable ordinal logistic regression was used to examine the relationship between suspected unintentional benzodiazepine consumption and DCS utilization frequency, categorized as: once, > once and < once/month, once/month, > once/month and < three times/month, weekly, and > weekly. Potential confounders included age, population centre size, and frequent unregulated opioid use (≥ weekly). In planned exploratory analyses, gender was included as a possible effect modifier.

**Results:**

Of 220 participants, 73 (33.2%) were women and 143 (65.0%) believed they had unintentionally consumed benzodiazepines in the previous six months. Bivariable ordinal regression showed a significant relationship between suspected unintentional benzodiazepine consumption and DCS utilization frequency (odds ratio [OR]: 2.11; 95% confidence interval [CI]: 1.22–3.69). However, after adjusting for confounders, particularly frequent unregulated opioid use, this association was no longer statistically significant (adjusted OR: 1.49; 95%CI: 0.75–2.99). We also failed to find that gender modified the relationship between suspected unintentional benzodiazepine consumption and DCS utilization.

**Conclusions:**

Findings suggest that frequent unregulated opioid use may best explain the frequency of DCS utilization. Although gender did not significantly modify the association between suspected unintentional benzodiazepine consumption and frequency of DCS utilization, continued research should explore the role and context of gender in this relationship.

## Background

In many settings internationally, the unregulated drug supply continues to evolve in harmful ways, as seen by the introduction of novel psychoactive substances. Fentanyl has proliferated in the opioid supply, supplanting heroin in many settings, while new concerns are emerging about the presence of xylazine and benzodiazepines in opioids and the broader unregulated drug market [13, 32, 46]. In Australia, benzodiazepines were involved in 45% of drug poisoning deaths in 2021, while etizolam (a benzodiazepine analog) was found in 60% of drug-related deaths in Scotland in 2020, a 19-fold increase from 2015 [8, 11, 27, 33],United Nations Office on Drugs and Crime, 2023). This trend extends to Canada where, in the province of British Columbia, the proportion of unregulated drug deaths involving benzodiazepines increased from 6.7% in 2019 to 48.3% in 2024 [4, 12]. In the United States more broadly, 16% of fatal opioid poisonings in 2020 involved benzodiazepines [20].

In addition to their effects on mortality, benzodiazepines can be prescribed as an anxiolytic and can cause a range of euphoric, hypnotic, and amnesic effects, with risks for dependence, agitation, aggression, and psychosis depending on the dose as well as individual and environmental contexts [7, 15, 18]. As central nervous system depressants, benzodiazepines can cause short-term side effects such as decreased respiratory rate, heart rate, and blood pressure as well as impaired judgment and loss of inhibitions and memory, particularly when combined with other drugs [7, 18]. Indeed, this slowing of critical body functions is compounded when benzodiazepines are consumed with other depressants such as opioids, increasing the risks of drug poisonings and other harms [6, 7, 32]. Withdrawal from benzodiazepines can occur after continued then reduced use, and is typically characterized by irritability, headaches, gastrointestinal upset, and insomnia, yet can also result in the development of more serious reactions such as perceptual disturbances (e.g., hallucinations) and seizures [7, 32, 44].

Risks associated with benzodiazepine use in the adulterated drug supply are not uniform across populations. In fact, literature from prior to the proliferation of benzodiazepines in unregulated drug supplies underscores that a disproportionate number of women die from the co-use of benzodiazepines and opioids [22, 25]. In an era with a high prevalence of benzodiazepine-adulteration in the unregulated opioid supply, many people who use drugs may be unintentionally exposed to benzodiazepines. In this context, some have suggested that the influx of benzodiazepines into the unregulated drug supply has been used to facilitate crimes such as robbery and assault, including sexual assault, noting that these crimes disproportionately impact women [16, 29, 31]. Therefore, it is important that harm reduction efforts consider this growing phenomenon of benzodiazepine-related harm as well as gender in their implementation.

There is a dearth of literature on links between unintentional benzodiazepine consumption among people who use drugs and harm reduction service utilization, including context and frequency of service uptake. Drug checking services comprise a harm reduction intervention that provides people with opportunities to find out the contents of their drugs, thus lowering the risk of unintentionally consuming substances such as benzodiazepines [1]. Given the increasing harms of benzodiazepines in the unregulated drug supply in British Columbia, the present study aimed to examine the relationship between suspected unintentional benzodiazepine consumption and frequency of drug checking service utilization. Moreover, considering the gendered context and impacts of unintentional benzodiazepine consumption, we conducted a secondary analysis that included gender as an effect modifier. We hypothesized that suspected unintentional benzodiazepine consumption would predict frequent drug checking service utilization given that people want to ensure their drug is not adulterated, particularly for women, who may be at heightened risk for pertinent harms.

## Methods

### Study context

This study is set in British Columbia, Canada, where government-sanctioned drug checking projects have been piloted increasingly since 2016, and where grassroots drug checking services are also offered through various methods (BC Centre on Substance Use, 2017). Point-of-care drug checking services throughout British Columbia rapidly adopted and now utilize Fourier Transform Infrared Spectroscopy (FTIR) machines alongside immunoassay strips (commonly referred to as 'test strips') due to their portability and specificity. FTIR machines can detect contents of a drug sample that are present in a concentration of 5% or greater, and since the FTIR does not use up the sample for analysis, it can be returned to the client, all within a ten-minute process (BC Centre on Substance Use, 2022; [40]). Benzodiazepine immunoassay strips are also adept at detecting the presence of benzodiazepines at lower thresholds, with a cut off of 300 ng/mL [5]. While fentanyl and benzodiazepine immunoassay strips are available for take-home use, this study is part of an ongoing, provincial drug-checking evaluation of 19 point-of-care drug checking services provided by staff throughout British Columbia (see Table 1 for sample population centre data), all of which use FTIRs and immunoassay strips [40].

**Table 1:** Socio-demographic data of respondents to a survey of drug checking service users in British Columbia, Canada (2021–2023) stratified by gender

	Gender		
	Total (%) n = 220	Men^1^ n = 144	Women^2^ n = 73
*Frequency of drug checking service utilization*			
Drug checking > once^3^	110 (50.0)	75 (52.1)	33 (45.2)
Drug checking once	110 (50.0)	69 (47.9)	40 (54.8)
*Suspected unintentional benzodiazepine consumption*			
Yes/Uncertain	143 (65.0)	92 (63.9)	50 (68.5)
No	77 (35.0)	52 (36.1)	23 (31.5)
Age (median, IQR)	46 (38 – 53)	47 (39.8 – 53)	43 (36 – 51)
*Population centre*			
≥ 100,000	197 (89.6)	130 (90.3)	64 (87.7)
< 100,000	23 (10.5)	14 (9.7)	9 (12.3)
*Frequent opioid use*			
≥ weekly	168 (76.4)	109 (75.7)	57 (78.1)
< weekly	52 (23.6)	35 (24.3)	16 (21.9)
*Suspected non-fatal drug poisoning*			
Yes/Uncertain	66 (30.4)	47 (32.6)	17 (23.3)
No	151 (69.6)	96 (66.7)	54 (74.0)

Data are based on the six months prior to participants’ interviews. Data are based on drug checking services that used both spectroscopy and immunoassay strips. Numbers may not add to the total due to missing responses. IQR = interquartile range.

1. This includes cisgender men (n = 144); no respondents were transgender men

2. This includes cisgender women (n = 71) and transgender women (n = 2)

3. Collapsed based on responses: > once and < once/month; once/month; > once/month and < three times/month; weekly; > weekly

### Data collection

Data for this study were derived from surveys with drug checking service users, administered between 2021 and 2023. As part of a larger drug checking evaluation, one main aim of the study was to examine factors associated with the frequency of drug checking service utilization. Surveys were constructed in collaboration with researchers with content expertise, people with lived experience, and other knowledge users as well as by using drug checking and related literature. A small group of participants piloted these surveys before recruitment expanded more widely.

Participants were eligible to be in the study if they were 14 years of age or older, had accessed drug checking services at least once, could communicate in English, and provided informed consent. Recruitment was facilitated by drug checking technicians who informed service users about the study, as well as through word-of-mouth. Surveys were administered by trained research assistants (n = 9), one of whom had living experience of substance use and drug checking service utilization. Most surveys (90.3%) were administered over the phone, while 9.7% were administered in person in Kelowna and Vancouver. Survey topics included perceptions of drug checking services, related behavioural intentions, substance use patterns, experiences with drug poisonings, and socio-demographic information. A $25 CDN honorarium was offered to each participant. The study received approvals from the University of British Columbia/Providence Health Care Research Ethics Board [H17-03158].

### Sample and variables of interest

The present analysis was restricted to participants who reported that they had not intended to use benzodiazepines in the previous six months. The outcome variable was the frequency of drug checking service utilization, based on the question, “In the last 6 months, how many times have you used drug checking services?” Responses were ordinally categorized as: once, > once and < once/month, once/month, > once/month and < three times/month, weekly, and > weekly. These categories were selected based on the distribution of responses participants provided to the survey question and to facilitate pragmatic application of the study findings in policy and practice. The exposure variable was suspected unintentional benzodiazepine use (yes or unsure vs. no), based on the question, “In the last 6 months, have you used any drugs that you now believe contained benzos but did not know ahead of time?” Responses were collapsed into “yes” and “not sure” vs. “no” as different benzodiazepines have different effects that may be masked by other substances, and it can be difficult for people to perceive their impairment when using benzodiazepines [14, 15]. However, we evaluated the robustness of this assumption by running a second analysis with the responses collapsed into "yes" vs. "not sure" and "no."

Several variables were included as possible confounders: age (per year older), population centre consistent with Statistics Canada [36] definitions (≥ 100,000 people vs. < 100,000 people), frequent opioid use (≥ weekly vs. < weekly), having a regular source to acquire unregulated drugs from (yes vs. no), and suspected drug poisoning in the previous six months (yes or unsure vs. no). In the context of this study, a drug poisoning was defined as experiencing negative effects from the unintentional consumption of a substance or more concentrated dose [45], based on the question "have you overdosed or had a negative reaction in the past 6 months?" Responses of yes and unsure were collapsed into one based on the unpredictability of the unregulated drug supply and the different ways, as well as frequency with which, people who use drugs experience undesirable effects [44]. Again, these responses were collapsed into "yes" vs. "no" and "unsure" in a second analysis to enhance the robustness of the study. We did not include data on previous detection of benzodiazepines because such data could have reflected a time when participants intentionally used benzodiazepines, were using drug checking services for someone other than themselves, or they may have experienced cross-contamination.

Consistent with our hypothesis, gender was included as an effect modifier. Gender categories were inclusive of cisgender and transgender individuals; however, only a small proportion (< 1%) of individuals self-identified as transgender, all of whom self-identified as women.

### Statistical analyses

Descriptive statistics were generated for the exposure and potential confounder variables stratified by the outcome variable. Then, bivariable and multivariable ordinal logistic regression analyses were performed to assess the relationship between suspected unintentional benzodiazepine consumption and frequency of drug checking service utilization. All confounders met the assumption of proportional odds except for regular source, which was removed from further analysis. To assess the independent relationship of suspected unintentional benzodiazepine consumption and frequency of drug checking service utilization, we constructed a multivariable model and adjusted for potential confounders in a stepwise fashion. First, we adjusted for age, population centre, and suspected drug poisoning in the previous six months. Next, we adjusted for frequent opioid use as a separate step based on the a priori assumption that it could mask and be a driver of how often people use drug checking services, particularly given the prevalence of benzodiazepine-adulterated opioids. Then, we added gender as an effect modifier to the multivariable ordinal logistic regression model to explore if it modified the relationship between unintentional benzodiazepine consumption and frequency of drug checking service utilization. All *p*-values are two-sided. All statistical analyses were performed using Statistical Analysis Software version 9.4 (SAS Institute Inc., 2023).

## Results

From the overall dataset (n = 233), a total of 220 respondents reported not intentionally using benzodiazepines and were included in this analysis. There were no statistically notable demographic differences between people who reported not intentionally using benzodiazepines and the rest of the dataset (data not shown). Of the 220 participants, 144 (65.5%) were cisgender men and 73 were (33.2%) cisgender and transgender women, with a median age of 46 years (interquartile range: 38 – 53; Table 1). Furthermore, 167 (76.3%) participants identified as white, 53 (24.2%) identified as Indigenous, and 11 (5.02%) identified as Black, East Asian, South Asian, Middle Eastern, and/or Latino. Most participants (n = 197 [89.6%]) resided in a population centre of 100,000 people or more in the previous six months. Of the overall sample, 168 (76.4%) participants frequently used opioids (≥ weekly). Additionally, drug checking services had been used more than once by 110 (50.0%) participants, and this frequency of service utilization was reported more amongst men (n = 75 [52.1%]) than women (n = 33 [45.2%]). Finally, 143 (65.0%) participants believed they had unintentionally consumed benzodiazepines in the previous six months, and this was reported more amongst women (n = 50 [68.5%]) than men (n = 92 [63.9%]).

**Fig. 1:**
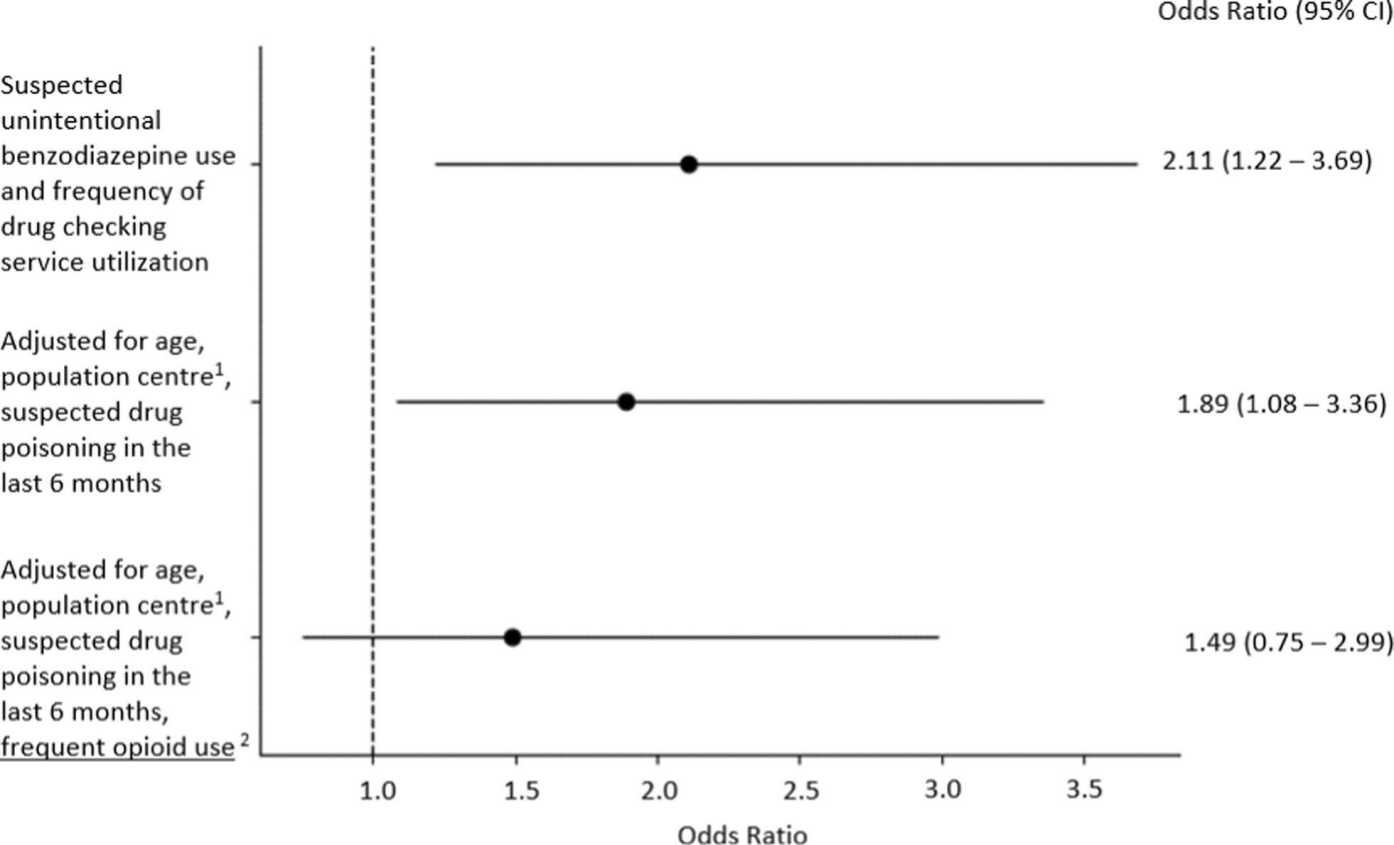
Plot of a multivariable ordinal regression model examining the relationship between suspected unintentional benzodiazepine consumption and drug checking services utilization frequency among respondents to a survey of drug checking service users in British Columbia, Canada (2021–2023), unadjusted and adjusted for confounders. *Note.* Data are based on the six months prior to participants’ interviews. Data are based on drug checking services that used both spectroscopy and immunoassay strips. Drug checking utilization frequency categorized as: once; > once and < once/month; once/month; > once/month and < three times/month; weekly; > weekly. 1. Categorized as population centres with ≥ 100,000 people and < 100,000 people 2. Using unregulated opioids ≥ weekly

Bivariable ordinal logistic regression showed a positive and statistically significant relationship between suspected unintentional benzodiazepine consumption and drug checking service utilization frequency (odds ratio [OR]: 2.11; 95% confidence interval [CI]: 1.22–3.69; Fig. 1; Table 2). This relationship remained significant after adjusting for age, population centre, and suspected drug poisoning in the previous six months in a multivariable ordinal regression analysis (adjusted odds ratio [AOR]: 1.89; 95% CI: 1.08–3.36). However, after adjusting for these same confounders and adding frequent opioid use, the relationship between suspected unintentional benzodiazepine consumption and frequency of drug checking service use was no longer statistically significant (AOR: 1.49; 95% CI: 0.75–2.99). In sensitivity analyses where we recategorized responses for suspected unintentional benzodiazepine use and suspected drug poisoning in the previous six months, we did not find significant differences with respect to the results (data not shown).

**Table 2:** Multivariable ordinal logistic regression analyses of factors associated with frequency of drug checking service utilization based on a survey of drug checking service users in British Columbia, Canada (2021–2023)

Variable	Adjusted odds ratio	95% confidence interval	*p* value
Main exposure variable			
Suspected unintentional benzodiazepine use (yes or unsure vs. no)	2.25	(0.76–7.39)	0.157
Secondary variables			
Age (in years)	1.03	(1.00–1.06)	0.029*
Frequent opioid use (≥ weekly vs. < weekly)	1.67	(0.76–3.78)	0.208
Population centre (≥ 100,000 vs. < 100,000)	1.73	(0.69–4.77)	0.264
Suspected non-fatal drug poisoning^1^ (yes or unsure vs. no)	1.42	(0.80–2.53)	0.226
Gender^2^ (men vs. women)	2.09	(0.73–6.67)	0.186

Data are based on the six months prior to participants’ interviews. Data are based on point-of-care drug checking services that used both spectroscopy and immunoassay strips

1. Based on the survey item "have you overdosed or had a negative reaction in the past 6 months?"

2. This includes cisgender men (n = 144), cisgender women (n = 71), and transgender women (n = 2).; no respondents were transgender men

To assess for possible effect modification by gender on the relationship between suspected unintentional benzodiazepine consumption and frequency of drug checking service use we added gender as an effect modifier to the multivariable ordinal logistic regression analysis (Table 2). We found that gender did not have a statistically significant effect on the association between suspected unintentional benzodiazepine consumption and frequency of drug checking service utilization (*p* > 0.05).

## Discussion

In this study with people who use drugs and who accessed drug checking services, two thirds (65%) of participants believed they had unintentionally consumed benzodiazepines. There appeared to be a significant relationship between suspected unintentional benzodiazepine consumption and drug checking service utilization frequency, yet the strength of this association diminished after adjusting for frequent unregulated opioid use. Effect modification analyses did not show an association between suspected unintentional benzodiazepine consumption and frequency of drug checking service utilization based on gender. It was not possible to determine if sample characteristics were representative of all people who use drugs in British Columbia, as these data are not available. However, a recent province-wide study using the British Columbia Harm Reduction Client Survey reported a similar gender distribution and slightly younger age amongst participants [38].

This provincial drug-checking evaluation study draws attention to, and contextualizes, the issue of benzodiazepine adulteration in the unregulated drug supply, alongside its impacts on the frequency of drug checking service use. A salient study finding is that the relationship between suspected unintentional benzodiazepine use and drug checking service utilization frequency is driven largely by unregulated opioid use, highlighting the nature and severity of benzodiazepine contamination in this drug supply. The significance of this issue is substantiated in a recent, mixed-methods provincial report documenting some of the ways in which people who use opioids in British Columbia have begun to adapt to this adulteration, and showing that self-reported use of benzodiazepines increased from 9.5% (n = 621) in 2019, to 20.7% (n = 537) in 2021 [44]. These findings on increases in the prevalence of intentional and unintentional benzodiazepine use among people who use drugs are also reflected in studies from other settings [11, 23, 30, 33, 37, 42], which, together, offer important evidence on the proliferation of benzodiazepines into and across unregulated opioid drug supplies.

Internationally, the growing trend of benzodiazepine contamination and associated harms, particularly in unregulated opioids, demonstrates an urgent need to invest in the implementation and evaluation of effective and accessible harm reduction programming, substance use disorder treatment, and drug policy interventions [8, 11, 20, 27, 33],United Nations Office on Drugs and Crime, 2023). With respect to drug checking services, FTIRs and immunoassay strips provide valuable data for informed drug use, yet these technologies are limited in their capacity to identify novel benzodiazepines consistently and accurately [23, 39]. Alternative and more recently available high-specificity technologies have been proposed, and strategies should be explored to examine ways that can better detect benzodiazepines [17, 23, 28, 44]. Drug checking services are not a panacea for addressing the drug poisoning crisis, however, and many people who use drugs do not frequent these services, which has been attributed to the inherent unpredictability of the drug supply and the lack of alternative options [9, 10, 19, 21, 23, 24, 26, 27, 35, 37, 44]. Addressing this crisis will indeed require a broader array of interventions including the scale-up of evidence-based treatment and harm reduction as well as the implementation and evaluation of novel strategies to address the root causes of the growing toxicity in illicit drug markets [43].

In exploratory analysis, this study did not find gender to be significantly associated with suspected unintentional benzodiazepine use and drug checking service use frequency, though the study may not have been adequately powered to do this analysis. Notably, this study did find a greater prevalence of unintentional benzodiazepine consumption amongst women but more frequent drug checking service utilization amongst men. Issues of statistical power and sample size are critical to note here given that the gendered dimensions of substance use and harm reduction are well substantiated in the extant literature and should not be discounted by the current study’s null findings. As one example, women who use drugs are known to face unique barriers to accessing harm reduction services, such as inequitable family responsibilities and gender-based stigma and discrimination, particularly for transgender, sexual minority, and/or racialized women (Godkhindi et al., 2022; Paschen-Wolff et al., 2023; United Nations Office on Drugs and Crime, 2023; Valasek & Bazzi, 2023). Future research should attend in depth and apply intersectional perspectives to the role and context of gender in the relationship between unintentional benzodiazepine consumption and frequency of drug checking service use, including to account for variegated risks and potential disparities in service uptake.

This study has several limitations. The sample was not randomly selected and therefore may not be representative of all people who use drug checking services in this setting. Because the data were based on self-report and did not include toxicology or drug checking service results, the experience of unintentional benzodiazepine consumption was based on the participants' perceptions. Nonetheless, self-report data hold value in applied public health research, and this study contributes to the relatively nascent drug checking literature. Additionally, the cross-sectional study design limited our ability to assess temporality. Future research should examine if unintentional benzodiazepine consumption immediately precedes suspected non-fatal drug poisonings, and if these drug poisoning experiences and potential confounder variables are associated with more frequent drug checking service use. There is also the potential for social desirability bias in this study, as with any survey on stigmatized issues. Finally, the small number of women in this study may be attributed to the study's recruitment methods in the context of inequitable harm reduction access barriers. Research leveraging gender-based and intersectional approaches may generate additional insights into the social context of drug checking service use and unintentional benzodiazepine consumption.

## Conclusion

The growing incidence of benzodiazepine contamination in the unregulated drug supply is increasingly causing harms and deaths internationally. Understanding how people who use drugs experience and attempt to reduce their risk of unintentional benzodiazepine contamination is critical to informing appropriate policy and practice responses, including in the context of drug checking implementation and adaptation, and with respect to drug market monitoring and evaluation of alternatives. The present study found that frequent unregulated opioid use, rather than previous suspected unintentional benzodiazepine use, may best explain one’s frequency of drug checking service utilization. The relationship between suspected unintentional benzodiazepine use and frequency of drug checking service utilization should be explored further, as should the role and context of gender in this relationship. For people who use drugs, particularly women with intersectional identities, providing and scaling up access to effective harm reduction measures such as drug checking services is needed to mitigate unintentional benzodiazepine consumption and associated harms.

## Data Availability

No datasets were generated or analysed during the current study.
