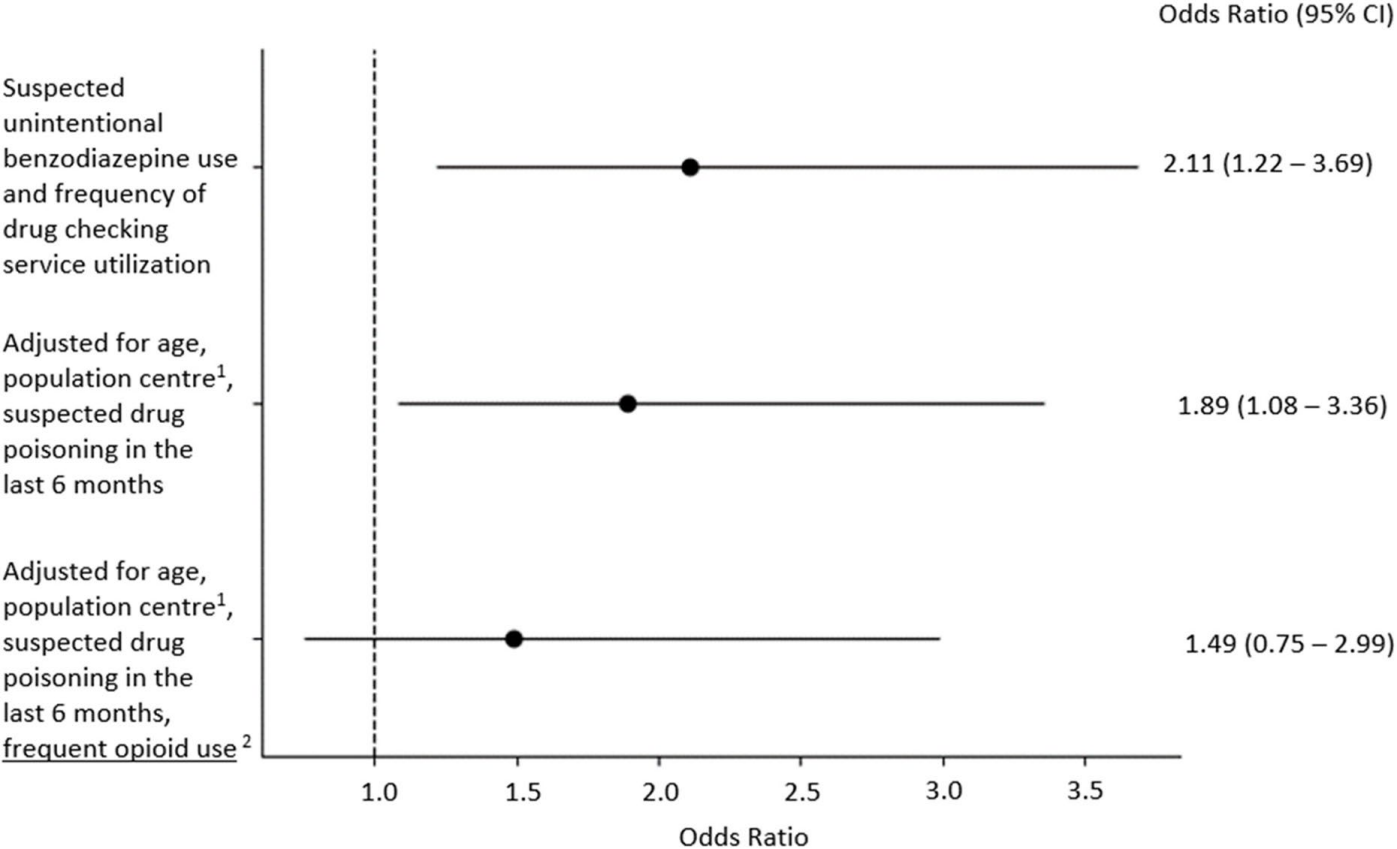# Correction: Unintentional benzodiazepine use and frequency of drug checking service utilization: a cross-sectional study

**DOI:** 10.1186/s12954-026-01497-9

**Published:** 2026-07-07

**Authors:** Lauren Airth, Trevor Goodyear, Brandon D. L. Marshall, Cameron Grant, Mark Lysyshyn, Susan G. Sherman, Evan Wood, Lianping Ti

**Affiliations:** 1 British Columbia Centre On Substance Use, 400-1045 Howe Street, Vancouver, BC V6Z 2A9 Canada; 2 School of Nursing, University of British Columbia, 1147 Research Road, Kelowna, BC V1V 1V7 Canada; 3 Campus Wellness and Education, University of British Columbia, 3272 University Way, Kelowna, BC V1V 1V7 Canada; 4 School of Nursing, University of British Columbia, T201-2211 Wesbrook Mall, Vancouver, BC V6T 2B5 Canada; 5 Department of Epidemiology, Brown University School of Public Health, 121 S Main St, Providence, RI 02903 USA; 6 Vancouver Coastal Health, 600-601 West Broadway, Vancouver, BC V5Z 4C2 Canada; 7 School of Population and Public Health, University of British Columbia, 2206 E Mall, Vancouver, BC V6T 1Z3 Canada; 8 Department of Health, Behavior and Society, Johns Hopkins Bloomberg School of Public Health, 624 N Broadway Street, Baltimore, MD 21205 USA; 9 Department of Medicine, University of British Columbia, 2775 Laurel Street, Vancouver, BC V6H 0A5 Canada

**Correction: Harm Reduct J 23, 36 (2026)**

**https://doi.org/10.1186/s12954-025-01381-y**

In this article [1], The Fig. 1 has been lost during final publication. The incorrect and correct versions of Fig. 1 are displayed below. The original article has been corrected.

Incorrect Fig. 1:


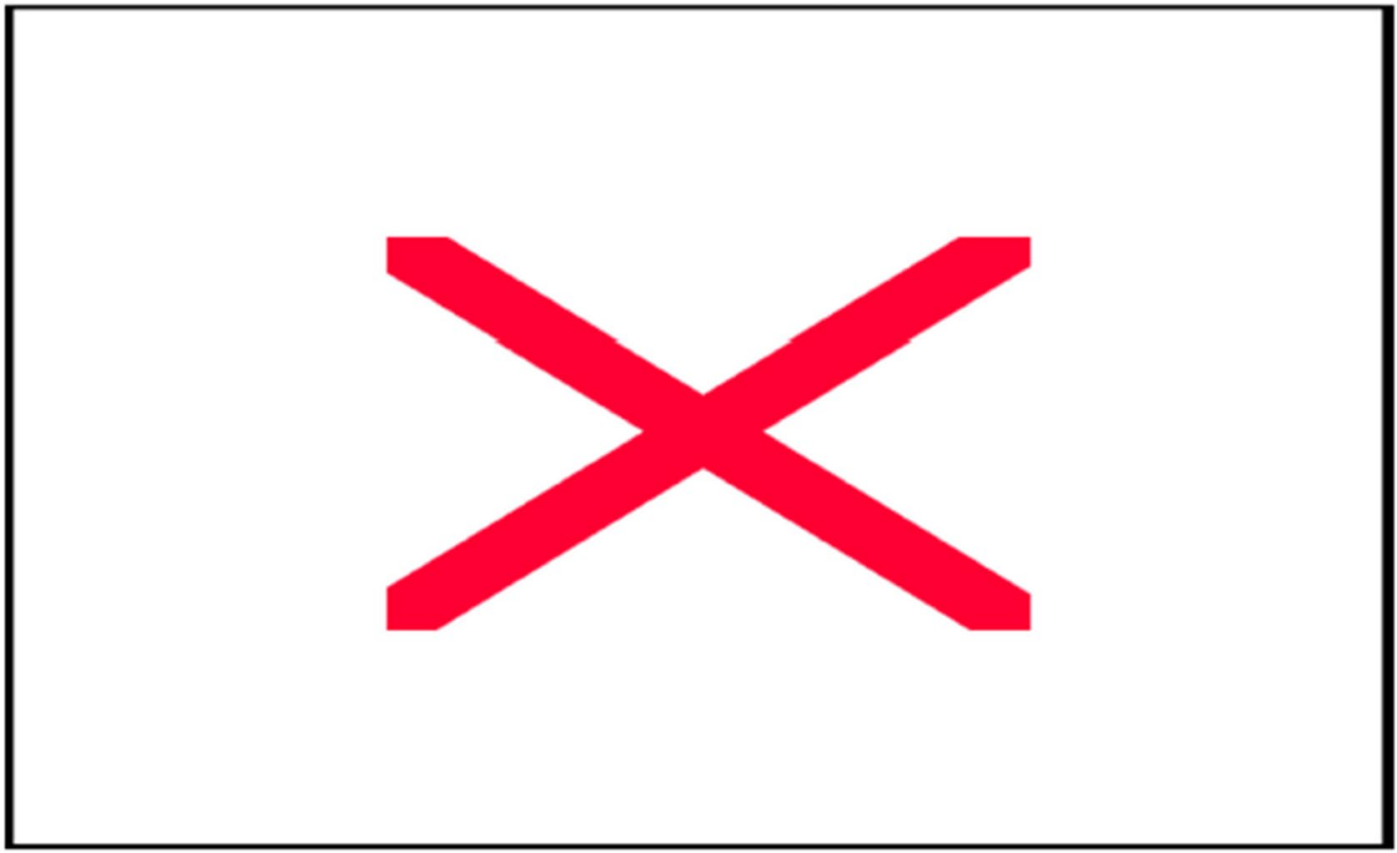


Correct Fig. 1: